# Public understanding of intersex: an update on recent findings

**DOI:** 10.1038/s41443-021-00485-w

**Published:** 2022-01-14

**Authors:** Peter Hegarty, Annette Smith

**Affiliations:** 1 The Open University, Milton Keynes, United Kingdom; 2 University of Surrey, Guildford, United Kingdom

**Keywords:** Human behaviour, Quality of life, Therapeutics

## Abstract

Surgical interventions on infants with intersex characteristics are considered justified by some on the grounds that they carry a high risk of intolerable stigma. However, public understanding of intersex and its medicalization are under-researched. We review recent qualitative and quantitative studies of the understandings of intersex and its medicalization among people who have no particular professional or public experience of intersex. First, such laypeople reason about clinical dilemmas by drawing on values in similar ways as expert healthcare professionals do. Second, laypeople can over-estimate the utility of current ‘umbrella terms,’ including *intersex*, for people with direct familial experience of intersex. Third, beliefs about good and bad effects of medical intervention are affected by framing intersex as either a medical condition or the natural basis for a social identity. Fourth, sexual identity is the best evidenced predictor of opinions about early surgical intervention and its legal limitation on human rights grounds. We argue that possible stigmatizing reactions from the public may not be a solid basis on which to justify early surgical intervention on intersex characteristics.

## Introduction: a lack of understanding

Psychosocial issues have long been assumed to be a reason that normalizing surgical interventions on intersex variations can be medically necessary.^1^ In particular, intersex variations have long been described as carrying such a risk of inevitable stigma that they call out to humanitarian medical practitioners who then “must do something” [1, 2]. And yet, as Liao and Simmonds (2014) and Sandberg, Pasterski, and Callens (2017) have noticed, there is precious little empirical research on stigma or social acceptance of people with intersex variations [3, 4]. This paper reviews six recent qualitative and quantitative empirical papers that aimed to address this gap in the literature (see Table 1). We first introduce this topic area.

**Table 1:** Samples, Methods, and Research Questions of Empirical Studies on Laypeople’s Understandings of Intersex.

Reference	Sample	Method	Research questions
Lundberg et al. (2019)	41 laypeople	Focus groups	How do laypeople resolve the dilemmas that emerge in clinical Practice with patients with intersex variations?
Lundberg et al. (2018)	41 laypeople, 33 parents & 22 young people	Focus groups (laypeople) & interviews (all others)	How and why do laypeople’s preferred terms differ among those with and without familial experience.
Hegarty et al. (2019)	99 psychology students	Randomized controlled experiment	How do freely available first-person narratives promoting social identity vs medical framing of intersex impact laypeople’s beliefs?
Smith & Hegarty (2021)	122 laypeople	Randomized controlled experiment	Does genital surgery on the clitoris appear to violate human rights more if the child is described as “intersex” rather than “female”?
Hegarty et al. (2021)	271 laypeople	Survey	What associations to umbrella terms for intersex bring to mind? What predicts support for medical vs social response to intersex?
Kingsbury & Hegarty (2021)	120 laypeople	Survey	What predicts support for medical vs social response to intersex? What analogies do first-person narratives bring to mind?

## Stigma is material to the bioethics of genital surgery on intersex variations

Stigma was framed as a central matter by intersex activists in the 1990s [5], and disagreement as to whether surgical interventions deliver people with intersex variations any relief from stigma is longstanding. In 19th century medicine, a search for such patients’ “true sex” characterized clinical investigation, and determining “true sex” of an apparent “hermaphrodite” through medical interpretation carried moral implications for the person’s sexual relationships and marriage [6, 7]. This moralization intensified with the medicalization of sexuality at the end of the 19th century [8, 9]. Even as some 19th century medical professionals developed surgical interventions to “correct” intersex variations, others critiqued their colleagues’ claims that surgery delivered this benefit [8]. In 19th century Europe, some adults with natural intersex variations were accepted by their families and communities, and their medical case histories imply fear about possible negative consequences of medical attention [7].

The boundary between beneficent and harmful medical intervention remains contested and morally drawn today. The current moral terrain is increasingly shaped by the interventions of major human rights bodies who describe unnecessary medical interventions as infringing human rights [10]. Whilst human rights statements assume a moral boundary between problematic interventions performed only for “cosmetic” or “normalizing” reasons, and those that are “medically essential” or “medically necessary,” contemporary healthcare professionals disagree amongst themselves about where to draw this such a moral line. Consider these two quotes from experienced healthcare professionals in European multi-disciplinary DSD (disorder of sex development) teams drawn from an interview study [11]. Both interviewees are referencing intervention on a hypospadic penis that presents no health risk per se, but which would, if not surgically altered, not allow the young male patient to urinate cleanly standing up. One cited a traditional, long-standing rationale (see also Griffiths, 2018a) that surgery might:

*“give the young person an opportunity to, you know, particularly as a boy to, you know, be continent to not least stand up at the urinal with his pals and pee in the pot at the same time”* [11, p. 107].

A second clinician presented an opposing, more contemporary, view:

“*the reality is that doesn’t happen, because children these days do not look at other people’s, other children’s penises, they just don’t do that. So, I’m not sure whether it’s medically essential”* [11, p. 107].

Does a boy really need to pee like his pals? Will his pals just look away if he cannot? These clinicians came to different answers to this question due to opposing psychosocial assumptions about the materiality of stigma in the lives of school-age boys, see also [12]. Both of their answers stand on shaky ground, and either clinician could, in good faith, pass on their beliefs to parents who might reasonably interpret those beliefs as medical expertise [13]. Whilst such parents bear the risks of stigma to their children in mind when making such decisions [14], neither clinician has the expertise in children’s social development to research what happens in situ at the urinal; training in pediatric urology does not provide this. If either were to search for relevant psychosocial literature, the best they will find are studies of parental reports rather than boys’ own accounts of their experiences [15, 16]. Children’s vulnerabilities in this context are obvious. Clinicians and parents are also less obviously threatened by obligations to wield medical authority and to make decisions in children’s best interests at critical morally charged junctures where there is less conceptual and empirical basis than there might appear to be.

## Researching the materiality of stigma

Since stigma was framed as a central matter by intersex activists in the 1990s, a number of researchers have investigated its effects in the lives of adults with intersex variations, finding that cultural norms of sexed embodiment and medically prescribed *silence* commonly impacted personal identity development by perpetuating shame [17, 18]. Kessler (1998) reported experiments in which young adult students with no particular experience of intersex conceptualized a decision regarding surgery on infants’ genitals from a parent or a child’s vantage point. Most opted for surgery from the parent’s perspective but refused it from the child’s perspective [19]. Streuli et al. (2014) randomly assigned medical students to contemplate a similar decision as a parent, whilst receiving either a medical or a psychosocial briefing about the child, leading 66% vs 23% of participants to opt for surgery by condition [20]. Clinical experts have called for research on minority stress in this arena (Lee et al. [21]); a psychosocial approach that examines how mental and physical health of individuals in minority groups are impaired by the dynamics of social stigma [22]. Stigmatization can occur in medical environments, as qualitative critical health research has repeatedly shown [23]. Recent studies using retrospective reports found that many, but not all people with intersex variations report stigma, that stigma unfolds within medical contexts and elsewhere, and that greater variation from normative sexed embodiment carries greater stigma risk [24–29]. Among the 1704 intersex-identified people who responded to the European Union Fundamental Rights Agency’s first survey that included intersex, more than one in three considered discrimination on the basis of their sex characteristics to be their biggest problem [30]. Although not always credited with the insight in this literature, early 1990s intersex activists were right to emphasize the importance of stigma.

The current paper adds to this picture by reviewing six recent studies on the group who are feared to be the stigmatizers of people with intersex variations: the general public. Table 1 lists the samples, methods, and research questions of these studies. We summarize these studies under five themes below.

### Wrestling with dilemmas

In Lundberg et al.’s (2019) ten focus groups 41 participants discussed three clinical dilemmas; (1) whether and how to assign gender to an infant whose genital anatomy did not signify either a male or female sex assignation, (2) whether to conduct genital surgery in infancy, and (3) whether and how to disclose to a girl at puberty that she had XY chromosomes [31]. This study was framed by an understanding that genuine dilemmas have no easy resolution, that wrestling with dilemmas requires extending existing common sense to the issue at hand, and that such thinking is often done in social interaction [32]. In the first context, participants considered both fairness and the risk of harm that would likely follow from making no gender assignation vs making a potentially incorrect one. Their discussions exposed uncertainty and opposing beliefs about the appropriateness of non-binary gender, whether gender was fundamentally biological or personal, and whether gender unfolded from within or was influenced from outside. The second dilemma leads participants to weigh both the extent of the anatomical variation from normality and the risks of both choosing surgery and rejecting surgery on damaging both the child and the adult, that the infant would later become. Behind these immediate concerns sat uncertainty about psychosocial questions. Could genital differences among growing children be socially accommodated or affirmed? How much should “normality” be valued for its own sake? Finally, whilst participants largely favored full disclosure to the girl with XY chromosomes, they struggled to agree on who should do the disclosing. Both healthcare professionals’ likely expertise and possible prejudices, and parents’ own emotions informed their arguments. This dilemma led to further discussion that when parents and their teenage children disagreed about healthcare pathways and gender identity, that both the young person’s right to self-determination and their protection from stigma were important concerns.

This study was part of the larger SENS research project, conducted in Scotland, England, Norway, and Sweden, which also included interviews with young people with intersex variations and their parents and healthcare providers. These diverse experts had wrestled with related dilemmas in real life, allowing a “thicker” description of the focus group data [33]. Like the healthcare professionals interviewed in the SENS project [11], focus group participants discovered that the logical foundations of their positions were surprisingly grounded in quite contestable common-sense beliefs [31]. Like the focus group participants, the healthcare professionals drew on such values as rights to care, self-determination, and avoiding harm. But these dilemmas still called for guesswork and exposed differences of opinion in both groups about social matters such as stigma, normality, and gender.

### Preferences and understandings regarding terminology

Lundberg, Hegarty and Roen (2018) compared the focus group participants’ preference for those terms with the preferences expressed in interviews by the young people and parents interviewed in the SENS project [34]. “Intersex” was the modal preference among focus group participants; 36.6% preferred it. However, only 7% of young people and parents interviewed in the SENS project preferred this term. “Disorders of sex development” was preferred by only 19.5% of focus group participants but by none of the young people or parents interviewed in the SENS project.

These figures are not surprising; larger surveys similarly show that medical terms are dis-preferred and that no umbrella terms are uniformly preferred among people categorized under those umbrella terms, or their family members (see Footnote 1). SENS interview and focus group participants also explained their preferences, and their explanations included information about what particular terms do and don’t do, and the contexts in which particular terms seemed useful or not. Focus group participants heard “intersex” as an affirmative term, but several experts by experience had not heard the term, whilst other experts by experience feared that it suggested psychological issues, including those pertaining to gender identity and sexual orientation. Experts by experience more often preferred descriptive terms that referred only obliquely to their variations. Laypeople may overestimate how useful linguistic terms, such as ‘intersex’ are as ways of managing a range of everyday contexts that experts by experience have really encountered and expect to encounter again in the future.

Finally, Hegarty, Donnelly, Dutton, Gillingham, Williams, & Vecchietti (2021) surveyed 271 UK and USA participants to understand the conceptual similarities and differences between controversial umbrella terms [35]. Participants gave free associations to three umbrella terms, “hermaphrodite,” “intersex,” and “disorders of sex development (DSD),” and reported familiarity with those terms. Content coding of their 1,457 responses showed conceptual similarities and differences in the three terms’ common meanings. Consistent with concerns that this term is dehumanizing, “hermaphrodite” produced more associations to plants, animals, and myths. Compared to “*DSD*” and “hermaphrodite”, “intersex” called to mind fewer references to biology and medicine and more references to gender minority adults and the gender binary. “Disorders of sex development” brought to mind more associations to biomedical issues and to children than did the other two terms. Because ethical dilemmas about infant genital surgery may trade off the risks of stigma to growing children vs grown-up adults, language choices may frame these risks by calling to mind images of children vs adults or images of bodies vs images of social identities.

### Framing medical harms and benefits

Carpenter (2018) has critiqued expert understandings to be limited by two dominant framings of intersex variations as (1) abnormalities to be normalized and (2) social identities akin to LGBT identities [36]. Abnormality and social identity have been long shown to be two common ways in which people frame a much wider range of physical traits that can occasion stigma [37]. Two experiments furthered our understanding of how one framing can be made dominant in a situational context. First, Hegarty, Smith, & Bogan-Carey (2019) randomly assigned psychology students to watch one of two videos that presented first-person narratives within either a narrative of medical normalization or a social identity narrative [38]. The medical video was a health promotion video from the UK National Health Service whilst the social identity video was created by Buzzfeed and Interact youth. Both videos were available on YouTube and both promoted themselves as addressing ignorance about intersex people. Students’ beliefs about intersex people and about the harms and benefits of medical intervention on intersex were assessed after watching one of the videos. A third baseline group watched no video.

Students greatly preferred the social identity video and its speakers over the medical video. Relative to baseline, the medical video significantly increased belief in the benefits of medicine for intersex people, whist the social identity video significantly decreased belief in medicine’s benefits and increased beliefs in medicine’s harms. The effect of the video was greatest among participants who scored lower on two proxy measures of intersex stigma; a social distance measure and a measure of belief in the binary gender. However, neither video produced a significant change in students’ beliefs that intersex people were normal or could experience full social inclusion, and neither video affected students’ curiosity to learn more about intersex. These first-person narratives shifted these students’ beliefs about medicine, but not their beliefs about intersex people as a group. In reality, the social identity video had been watched on YouTube many more times than the medical narrative video. Both those view counts and the controlled experiment suggested the increasing popularity of a social identity framing of intersex as opposed to early surgery on intersex variations.

Second, Smith and Hegarty’s (2021) participants were randomly assigned to read a short text about clitorectomies on infants described either as ‘intersex’ or ‘female’ [39]. Participants indicated their levels of agreement that genital cutting infringed human rights using questionnaire items based on statements excerpted from human rights documents. Overall, women considered clitorectomy more violating than men did. As predicted, clitorectomy was judged to violate human rights significantly more when the child was described as ‘female’ than ‘intersex.’ Two psychological variables were found to moderate this framing effect. First, its effect was greatest among participants who trusted medical authority the most. Second, its effect varied by scores on the conservatism subscale of the right-wing authoritarianism (RWA) scale, which measures the willingness to obey societal authorities. Specifically, RWA conservatism scores were negatively correlated with the belief that the clitorectomy violated human rights only when the infant was described as “female.” Genital cutting on a child described as “intersex” may seem particularly tolerable among laypeople who trust medicine the most and who are willing to obey conservative sources of authority the most.

### Predicting belief about medical and social responses

**Fig. 1:**
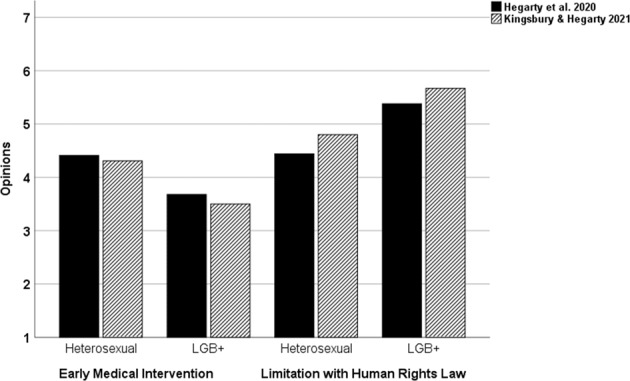
Different Opinions about Medical Intervention and its LImitatation Between Heterosexual- and LGB+-Identified Participants in Two Published Studies.

In Hegarty et al.’s (2021) survey, the UK and USA participants reported their opinions towards six responses to people with intersex variations and their families: (1) early medical intervention, (2) sympathy with their parents’ feelings, (3) belief in the power of effective parenting, (4) endorsement of support groups, (5) legal prohibition on medical intervention, and (6) social equality recognition [35]. Endorsement of opinions 2–6 were all positively correlated with each other and all were negatively correlated with the endorsement of early medical interventions, confirming opinion that medicine and social identity are the currently dominant framing of intersex [36]. Opinions about these six responses did not covary systematically by participants’ nationality, gender, age, ethnicity, religion, educational attainment, social class, parental status, or experience of healthcare work. However, heterosexual-identified participants endorsed medical intervention significantly more, whilst LGB+ participants endorsed responses 2–6 significantly more. These sexual orientation differences were replicated by Kingsbury and Hegarty [40] with respect to opinions 1 and 5 [40] (see Fig. 1).

Both studies examined psychological variables that might moderate and explain this sexual orientation difference. Group differences in belief in the gender binary [35, 40] right-wing authoritarianism [35], and strength of social identity [40] moderated the difference. This last finding bears expansion. Specifically, identification with one’s sexual orientation in-group significantly predicted heterosexual laypeople’s greater support for medical intervention and greater opposition to its legal limitation in Kingsbury and Hegarty (2021). Among LGB+ participants in the same study, identification with one’s sexual orientation in-group was not significantly correlated with these opinions. LGBT organizations have been largely supportive of human rights-based limits on surgical intervention on intersex variations and consequently have been critiqued by some medical experts and advocacy groups as projecting identity-based concerns where they do not belong [41]. However, the available this evidence suggests that it is heterosexual-identified laypeople, and not LGB+ laypeople, whose identification with their sexual orientation might determine how they think about medical intervention and its legal limitation in this domain.

### Generating analogies to intersex

Finally, Kingsbury and Hegarty [40] also asked participants to read three interview extracts from Davis’ (2015) award-winning sociological monograph Contesting Intersex, which Davis had conducted with a medical professional, a parent, and an adult with an intersex trait [42]. After reading these three interview extracts, Kingsbury and Hegarty (2021)’s participants listed up to three analogies to intersex, which were classified into 11 content categories. The most common analogies were those that referred to medicalized contexts, accounting for 24% of all analogies produced. Participants who produced analogies that were classified into a greater number of the content categories also tended to endorse binary gender beliefs less, to support medical intervention less, and to support its legal prohibition more. Laypeople who brought less diverse thoughts to mind in response to reading about intersex most strongly endorsed surgical intervention.

## Synthesis and clinical recommendations

What general lessons can be drawn now? First, lay people’s understandings of intersex and its medicalization can be researched using ordinary social science methods; focus groups, interviews, experiments, and surveys. In the course of this work, we heard experts express doubt that this work could be done ethically and with validity. However, anonymous survey respondents usually complimented our efforts upon full debriefing. Structural stigma can result in fear and avoidance of a group, leading social researchers to avoid studying the stigma experienced by that group [43]. If structural stigma has led others to fear conducting research on the public understanding of intersex, we hope to have chipped away at those fears here, whilst responding to calls to develop research on stigma in this area [3, 4]. Our studies lead us to several conclusions that address questions often asked in larger debates about intersex variations and the medical interventions that they can prompt.

First, Second, is there a clear bioethical line between (good) medically necessary and (bad) cosmetic interventions on intersex variations [11]? Laypeople, like health care professionals, can and do ground their bioethical reasoning about what is best to do when intersex variations are presented in clinical contexts in psychosocial concerns that are matters of debate and disagreement [11, 31]. A single essential clear line may be assumed to exist, but many such lines may be drawn in response to dilemmas. As such, these findings resonate with philosophical arguments that essentialist categories cannot ground bioethics in this area [44], and historicist argument that contemporary investment in the promise that genetics will not ‘sort out’ bioethical questions [45]. Elsewhere this approach has been called strategic anti-essentialism [46].

Third, to whom—among the public–should a young person or their anxious parent turn for support? There are diverse views among the general public about whether surgical intervention on intersex characteristics and other social forms of support are good and viable things [35]. Sexual orientation is the clearest predictor of variation in opinions about both medical and social responses [35, 40]. In marked contrast, neither parental status itself nor healthcare experience predicted laypeople’s (1) sympathy with parents, (2) belief in their parenting power, (3) endorsement of support groups nor (4) social equality recognition for intersex adults [35]. However, someone who is LGB+-identified is statistically more likely to support and to be optimistic about all four social responses. Concerns that LGB+ people project identity concerns onto this domain [41] might be diverted ask whether heterosexual social identity might affect beliefs about early surgery on intersex characteristics [40]. We note also that sociologists have highlighted concerns that anti-gender campaigns which target LGBT rights may be limiting developments in intersex care [12, 47].

Fourth, can people with intersex variations hope to reduce stigmatization by sharing their personal narratives with others? Laypeople’s opinions about the medicalization of intersex, and surgical interventions on infants in particular, are not completely crystallized. Those opinions can be affected by framing intersex as either primarily a medical or psychosocial issue, with consequences for beliefs about whether genital surgery in infancy is benevolent or not [20, 38]. Sharing experiences may be a form of ‘contact’ that reduces prejudice toward intersex people as it reduces prejudice in many other areas [48]. Laypeople who find first-person accounts about intersex to be thought-provoking maybe those who endorse medical intervention the least and support its legal limitation the most [40]. However, sharing one’s own experience as an intersex person seems to impact laypeople’s beliefs about the harms and benefits of medical intervention more reliably than it impacts beliefs about intersex people as a group [38].

Third, Fifth, several intersex rights organizations who call for rights to self-determination and so oppose early surgical interventions have described it as problematic that intersex and transgender groups are confused in the public mind. We found empirical evidence that this concern is grounded when we asked laypeople to reason about clinical dilemmas, to report what umbrella terms bring to mind and to draw analogies [31, 35, 40]. Relatedly, participants who endorsed gender binary beliefs, that would negate the existence of transgender and non-binary people, endorsed surgical interventions on children’s genitals less [35, 39, 40] and were more open to influence from first-person narratives [38]. These findings have implications for intersex advocacy groups. Whilst many laypeople do consider intersex and transgender to be analogous (or wrongly consider them identical), those laypeople who are most likely to support intersex human rights are also those who endorse the gender binary the least. Because transgender appears to be the more familiar concept, these findings also imply that research on public understanding of intersex should clearly tease apart beliefs and opinions about transgender and intersex groups separately. Some researchers have done this [48]. Others have not [49]. Whilst both approaches have merits, we doubt that research that does not actively tease these concepts apart will yield distinct insights about the public understanding of intersex.

## Concluding thought

At the outset, we contrasted two clinical experts in specialized DSD teams who had different views about medical necessity grounded in different assumptions about the material risk of social stigma brought about by a hypospadic penis. Our work suggests that members of the public may similarly be torn between two paradigms; traditional medical authority vs. openness to rethinking what is necessary given changing societal norms around sex characteristics and their relationship to gender norms. This vision of the public stands at odds to the traditional one in which naturally occurring intersex variations are assumed to inevitably invite unbearable stigma such that surgical intervention on those traits must be the most benevolent response to them. Regarding the stigma of hypospadias to young boys, Morland (2001), who experienced such an intervention, concluded that when a child is at risk of being bullied that the problem might be better located in the social dynamics of bullying than in the physical trait that draws the bully’s attention [50]. Twenty years on from his letter to The Lancet on these points, educational professionals are showing greater interest in developing the competency for forestalling the effects of stigma on children with intersex variations in their care [51, 52]. Because of historical investment in medical authority to singularly provide it, the care and flourishing of children with variations in sex characteristics have become managed such that the scalpel has been asked to do the work of the soccer coach, the primary school teacher, and the social worker. We do not doubt that stigmatization of people with intersex variations is a real issue but would urge that our medical colleague’ ethics should be informed by an understanding of it as a variable experience, and one that—for almost two centuries—some medical authorities have doubted whether stigma is a warrant for surgical normalization. We are aware that this reframing of the relationship between stigma and medical necessity may create cognitive dissonance among healthcare professionals torn between traditional medical authority and openness to the input of other disciplines and professions. But that is not the most important issue at hand.

Footnote.

1. Throughout we use intersex to refer to physical attributes that differ from typical norms of male and female embodiment, and to people who identify with that term. We are aware that some feel that this term has been imposed on them by others and that the term “disorder of sex development” has long been preferred in medical professions where it was assumed to be less pejorative and clearer [53, 54]. (Pasterski, Prentice & Hughes, 2010) (Lee, Houk, Ahmed, & Hughes, 2006). Empirical studies of patients’ preferences across three continents show no umbrella term to be preferred by a majority, a clear dislike of pathologizing language, and preferences to refer to specific variations rather than to use ‘umbrella terms’ [55–61]. Cogent interdisciplinary communication often requires the use of imperfect linguistic tools with explicit recognition of their limitations and readers’ good faith.
